# Vagal pathways and heart rate variability in recovery after caesarean delivery: a framework for multimodal autonomic-immune phenotyping

**DOI:** 10.3389/fphys.2026.1935418

**Published:** 2026-08-14

**Authors:** Yi Zhang, Shuang Guo, Qingjun Zeng, Haishan Cui, Chunmei Li

**Affiliations:** Department of Anesthesiology, Wanzhou District Maternal and Child Health Hospital, Chongqing, China

**Keywords:** autonomic nervous system, caesarean delivery, cholinergic anti-inflammatory pathway, heart rate variability, neuromodulation, postoperative recovery, vagus nerve

## Abstract

Recovery after caesarean delivery involves interacting inflammatory, autonomic, neuroendocrine, nociceptive and psychosocial processes. This Mini Review examines whether vagal pathways and heart rate variability (HRV) can provide a mechanistic and measurable framework for studying that recovery. The inflammatory reflex and cholinergic anti-inflammatory pathway offer a biological rationale: neural signals can restrain cytokine production through an autonomic-immune circuit that includes splenic sympathetic signalling, norepinephrine-responsive acetylcholine-producing T cells and the α7 nicotinic acetylcholine receptor (α7nAChR) on macrophages. However, the anatomy and relative vagal and sympathetic contributions to this circuit remain debated. HRV, particularly the root mean square of successive differences and high-frequency power under standardized recording conditions, indexes cardiac parasympathetic modulation rather than whole-body vagal activity or splenic anti-inflammatory output. Pregnancy is accompanied by substantial autonomic adaptation, and postpartum HRV follows a dynamic trajectory, but existing longitudinal data do not establish when individual values return to pre-pregnancy baselines. In obstetric anaesthesia, HRV has mainly been studied as a preoperative predictor of spinal anaesthesia-induced hypotension; evidence for serial HRV as a recovery measure remains limited. We therefore propose a testable framework in which standardized longitudinal HRV is paired with inflammatory biomarkers, patient-reported recovery and clinical milestones after caesarean delivery. Non-invasive interventions such as transcutaneous auricular vagus nerve stimulation, slow-paced breathing and HRV biofeedback are plausible probes of this framework, but their target engagement and clinical effects require rigorous sham-controlled evaluation. HRV should be treated as a candidate component of multimodal recovery phenotyping, not as a direct measure of the cholinergic anti-inflammatory pathway.

## Introduction

Caesarean delivery is one of the most common major operations worldwide, and its use has increased across regions and income settings (Betrán et al., 2016; Boerma et al., 2018). Recovery is commonly assessed using patient-reported instruments such as the Obstetric Quality of Recovery-11 (ObsQoR-11), together with clinical milestones including mobilisation, oral intake, return of bowel function and readiness for discharge (Ciechanowicz et al., 2019). Enhanced Recovery After Caesarean programmes have improved the organization of perioperative care, but objective physiological characterization of maternal recovery remains limited (Sultan et al., 2020; Caughey et al., 2026).

Recovery after surgery is not a single biological process. It reflects interactions among tissue injury, inflammation, autonomic and endocrine stress responses, nociception, sleep, mobility and psychological wellbeing. Within this network, vagal pathways are of particular interest because they influence cardiac regulation, gastrointestinal function and neuroimmune signalling. The inflammatory reflex and the cholinergic anti-inflammatory pathway provide a plausible mechanism through which neural activity can limit excessive cytokine production after tissue injury (Borovikova et al., 2000; Tracey, 2002; Wang et al., 2003).

Heart rate variability (HRV) may offer a non-invasive window on one component of this physiology. The central question is not whether HRV directly measures inflammation or the vagus nerve, because it does not. Rather, the question is whether serial, standardized indices of cardiac autonomic modulation can contribute to a multimodal phenotype of recovery after caesarean delivery. This distinction matters clinically: an inexpensive ECG-derived signal could be attractive for repeated perioperative measurement, but convenience does not establish construct validity. Any proposed biomarker must show that it adds information beyond heart rate, routine observations and patient-reported outcomes.

This Mini Review integrates HRV physiology, the debated anatomy of the inflammatory reflex, pregnancy-related autonomic adaptation and emerging neuromodulation strategies, while keeping the distinction between a cardiac proxy and immune effector signalling explicit. The aim is not to declare a validated biomarker or treatment target, but to define a coherent physiological model, identify where its assumptions may fail and specify studies capable of testing it.

Our group has published a scoping review protocol that maps the clinical HRV literature after caesarean delivery within a recovery-assessment framework (Zhang et al., 2026). The present Mini Review addresses a distinct question: it evaluates the physiological coherence and limitations of linking cardiac HRV with autonomic-immune signalling and does not report results from that review.

## Literature approach

A purposive PubMed search was updated in July 2026 using combinations of terms related to caesarean delivery, pregnancy, postpartum physiology, heart rate variability, vagus nerve, inflammatory reflex, cholinergic anti-inflammatory pathway, postoperative recovery, transcutaneous auricular vagus nerve stimulation and HRV biofeedback. Foundational mechanistic studies, primary obstetric and perioperative studies, measurement guidance and recent reviews were prioritized. This was not a systematic review; the literature was selected to examine the coherence, limitations and testability of the proposed physiological framework.

## Heart rate variability as an index of cardiac autonomic modulation

HRV describes variation in consecutive normal-to-normal cardiac intervals. Time-domain indices include the root mean square of successive differences (RMSSD), which is commonly used as an index of short-term vagally mediated cardiac modulation, and the standard deviation of normal-to-normal intervals (SDNN), which reflects total variability over the recording period. In frequency-domain analysis, high-frequency (HF) power is strongly influenced by respiratory sinus arrhythmia and parasympathetic modulation. Low-frequency (LF) power has mixed physiological determinants, and the LF/HF ratio should not be interpreted as a direct measure of sympathovagal balance (Task Force of the European Society of Cardiology NASP and Electrophysiology, 1996; Billman, 2013; Shaffer and Ginsberg, 2017).

Interpretation depends on acquisition conditions. Respiration, posture, time of day, activity, pain, temperature, sleep, anaemia and drugs used during neuraxial anaesthesia and postoperative care can materially alter HRV. HF power is especially sensitive to breathing frequency and tidal pattern. Accordingly, studies of maternal recovery should prespecify ECG acquisition, artefact handling, recording duration, posture, respiratory conditions and the interval from analgesic, vasoactive or antiemetic medication. Neuraxial anaesthesia should also be treated as a distinct, time-varying autonomic perturbation: spinal block can change HRV within minutes (Kawamoto et al., 1993), while block height, spinal or epidural technique, local-anaesthetic and neuraxial-opioid doses, vasopressors, anticholinergic drugs, sedatives and uterotonics may affect intraoperative and early postoperative recordings. Future studies should standardize the technique where feasible, record these exposures prospectively, and use prespecified post-dose exclusion windows or time-varying covariates. HRV provides information about sinus-node autonomic modulation under defined conditions; it is not a direct recording of vagal nerve traffic and should not be used as a stand-alone measure of systemic autonomic state.

Metric selection should follow the biological question. RMSSD is relatively suitable for short recordings and is less dependent on recording duration than SDNN, whereas HF power requires careful treatment of respiration and spectral methodology. SDNN reflects combined sources of variability and cannot be labelled a selective vagal measure. Mean heart rate should be reported because HRV depends mathematically and biophysically on heart rate, which can distort between-person and longitudinal comparisons (Monfredi et al., 2014). Non-linear measures, including Poincaré-derived SD1 and SD2, sample or approximate entropy, and detrended fluctuation analysis, have been examined in perioperative populations, but their reproducibility and incremental value in short postpartum recordings remain unestablished. A small, prespecified set of interpretable metrics is preferable to exploratory reporting of many correlated indices.

## The inflammatory reflex and the splenic anti-inflammatory circuit

The inflammatory reflex describes neural sensing and regulation of immune activity. Peripheral inflammatory signals can reach the brain through vagal and non-vagal afferent pathways, and central autonomic networks can then influence peripheral cytokine production (Tracey, 2002). Experimental vagus nerve stimulation reduced circulating tumour necrosis factor during endotoxaemia, while acetylcholine suppressed macrophage cytokine release through the α7 nicotinic acetylcholine receptor (α7nAChR) (Borovikova et al., 2000; Wang et al., 2003).

**Figure 1:**
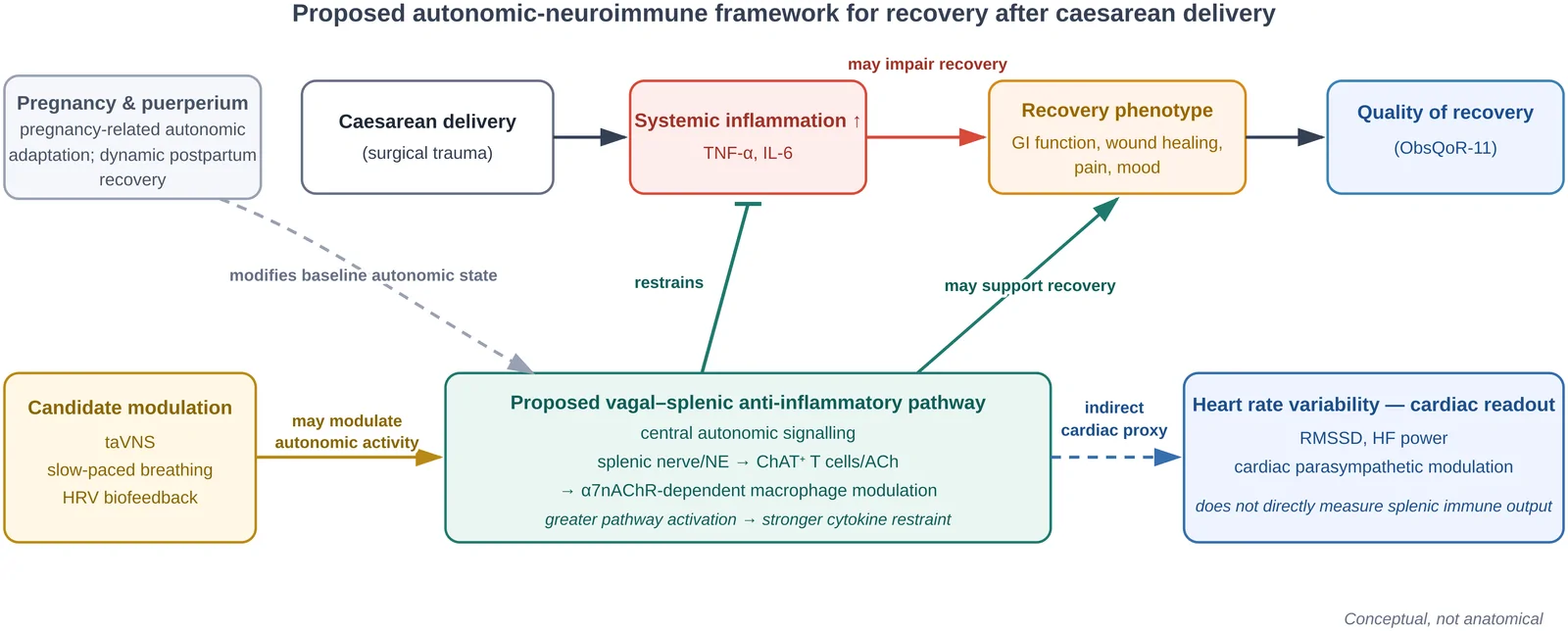
Proposed autonomic-neuroimmune framework for recovery after caesarean delivery. Caesarean delivery induces systemic inflammation, which may impair gastrointestinal function, wound healing, pain, and mood and thereby reduce quality of recovery. A proposed vagal-splenic anti-inflammatory pathway may restrain cytokine activity and support recovery. The central-to-splenic relay and α7 nicotinic acetylcholine receptor (α7nAChR)-dependent macrophage modulation are represented conceptually and should not be interpreted as established pathways in postpartum patients. Pregnancy-related autonomic adaptation may modify the baseline autonomic state. Transcutaneous auricular vagus nerve stimulation (taVNS), slow-paced breathing, and HRV biofeedback are candidate modulators rather than established therapies. Heart rate variability (HRV), represented by RMSSD and HF power, is a cardiac parasympathetic readout and an indirect proxy rather than a direct measure of splenic immune output. Solid arrows indicate directional relationships, the T-bar indicates restraint, and dashed arrows indicate contextual modification or an indirect proxy. The diagram is conceptual and not anatomical.

The efferent pathway is more complex than a direct vagal projection to splenic macrophages. In experimental models, the splenic sympathetic nerve is required for suppression of tumour necrosis factor, and norepinephrine released in the spleen activates β2-adrenergic receptors on choline acetyltransferase-positive T cells. These lymphocytes release acetylcholine, which acts on α7nAChR on macrophages (Rosas-Ballina et al., 2008; Rosas-Ballina et al., 2011). Martelli et al. argued more specifically that vagal stimulation does not evoke action potentials in the splenic sympathetic nerve and that a simple vagal-splenic efferent pathway does not explain the inflammatory reflex response to endotoxaemia (Martelli et al., 2014). Later anatomical and physiological reviews likewise describe splenic neuroimmune regulation as context dependent and potentially mediated by multisynaptic vagal-splanchnic interactions rather than direct vagal innervation of the spleen (Bassi et al., 2020). **Figure 1** therefore represents the central-to-splenic connection as a proposed multisynaptic autonomic relay. The illustrated splenic circuit is derived principally from experimental models and should not be interpreted as an established pathway in postpartum patients.

This circuit is relevant to abdominal recovery, but most causal evidence is preclinical. In a mouse model of postoperative ileus, vagal stimulation or α7nAChR activation reduced intestinal muscular inflammation and improved gastric emptying (Bonaz, 2007; The et al., 2007). The clinical phenotype after caesarean delivery is also shaped by neuraxial opioids, systemic analgesics, early feeding, mobilisation and obstetric factors, so delayed bowel function cannot be attributed to one neuroimmune pathway. These findings support biological plausibility for effects on gastrointestinal recovery, but they do not establish that cardiac HRV tracks this immune circuit or that the pathway determines global recovery after caesarean delivery.

The afferent and efferent components should also be separated. Vagal afferents are one route by which visceral inflammatory information reaches the brainstem, but circulating mediators, spinal afferents and humoral signalling also contribute. Similarly, a change in cardiac parasympathetic modulation may occur without a parallel change in splenic sympathetic activity. **Figure 1** therefore treats inflammation, cardiac HRV and clinical recovery as linked measurement domains whose temporal relationships require empirical validation.

## Autonomic adaptation during pregnancy and the postpartum period

Pregnancy produces substantial cardiovascular and autonomic adaptation. Resting heart rate generally rises and several HRV indices decline across gestation, although trajectories vary by gestational stage, measurement method and population (Kuo et al., 2000). Wearable monitoring across pregnancy and the first three postpartum months showed a postpartum fall in heart rate and increases in several HRV indices (Sarhaddi et al., 2022a). Importantly, that study did not measure individual pre-pregnancy baselines; it therefore could not establish whether autonomic indices had returned to pre-pregnancy values by three months. The trajectories were derived from Samsung Gear Sport wrist photoplethysmography. In a separate validation study in healthy adults, HRV accuracy was acceptable during sleep, but most indices showed high errors during awake recordings, and postpartum-specific validity has not been established (Sarhaddi et al., 2022b). These wearable trajectories should therefore not be considered equivalent to standardized ECG-derived HRV.

Mode of birth, labour exposure, haemorrhage, sleep disruption, breastfeeding, pain and postpartum medication may all modify the early trajectory. Breastfeeding should not be treated only as a nuisance covariate: infant suckling has been associated with lower maternal heart rate and greater parasympathetic HRV than comparable mother-infant contact without suckling (Ohmura et al., 2023). Feeding status and the interval since breastfeeding should therefore be recorded as biologically meaningful, time-varying exposures, while oxytocin remains a hypothesized rather than proven mediator. Existing wearable studies have mainly examined stress, mental health and broad perinatal trends rather than standardized perioperative recovery after caesarean delivery (Byfield et al., 2025). Evidence that lower prepartum RMSSD predicts later depressive symptoms is exploratory and does not support HRV as a stand-alone screening test for postpartum depression (Solorzano et al., 2022). Nevertheless, the convergence of surgical stress with pregnancy-related autonomic adaptation makes caesarean delivery a useful setting in which to study dynamic autonomic recovery.

The relevant baseline is therefore not a generic healthy non-pregnant value. A measurement obtained before neuraxial anaesthesia reflects late-pregnancy physiology, anticipation, fasting, positioning and any preceding labour. Postoperative measurements reflect rapid changes in volume status, pain, medications, maternal-infant contact and sleep. Interpretation should focus on within-person trajectories and clinically defined subgroups rather than comparison with universal HRV reference ranges.

## Current perioperative and obstetric evidence

Outside obstetrics, lower preoperative HRV has been associated with perioperative complications, although studies differ substantially in populations, recording methods and outcomes (Frandsen et al., 2022). In patients undergoing lung cancer surgery, preoperative HRV indices were associated with postoperative pneumonia and subsequent lung-function recovery, but the observational design cannot establish vagal causation (Lai et al., 2025). Associations between HRV and postsurgical pain have also been reported, with important confounding by respiration, medication, physical activity and baseline health (So et al., 2021). These studies primarily address prediction; they do not validate HRV as a serial measure of recovery.

In obstetric anaesthesia, the dominant application has been prediction of hypotension after spinal anaesthesia. Baseline HRV, including LF/HF-derived measures and composite autonomic indices, has shown variable predictive performance (Hanss et al., 2005; Jendoubi et al., 2021; Yu et al., 2021). This literature demonstrates feasibility of preoperative HRV acquisition but offers little evidence about maternal autonomic trajectories over the hours and days after delivery. It also reinforces the need to avoid physiological overinterpretation of LF/HF.

Prediction and recovery assessment are different use cases. A baseline variable can predict a complication without changing in proportion to recovery, while a useful longitudinal marker must be responsive to change, reproducible under repeated measurement and associated with outcomes over time. Validation after caesarean delivery therefore requires serial data rather than extrapolation from the hypotension-prediction literature.

## A testable framework for recovery after caesarean delivery

A cautious working model can now be stated. Caesarean delivery produces tissue injury, nociceptive input and inflammatory activation. Central autonomic networks respond to these signals and may influence gastrointestinal, cardiovascular and immune regulation. Serial HRV may capture part of the cardiac autonomic response, whereas inflammatory biomarkers capture a distinct downstream domain. The two may covary, but neither should be treated as a surrogate for the other without validation.

The model generates testable predictions. First, trajectories of RMSSD and HF power obtained under standardized conditions should be examined against ObsQoR-11 scores, time to mobilisation, gastrointestinal recovery, analgesic consumption and sleep. Second, temporal coupling between HRV and biomarkers such as interleukin-6, C-reactive protein or tumour necrosis factor should be quantified rather than assumed. Biomarker sampling should follow marker-specific kinetics: interleukin-6 may peak early after surgery, C-reactive protein later over 48–96 hours and circulating tumour necrosis factor may be inconsistently detectable (Kragsbjerg et al., 1995). HRV-biomarker coupling should therefore use prespecified marker-specific windows rather than a common schedule. Third, elective and emergency caesarean delivery should be analysed separately because labour duration, catecholamine exposure, infection risk and urgency produce different physiological starting points. Finally, breastfeeding, anaemia, haemorrhage, vasoactive drugs, neuraxial opioids, systemic analgesics and respiratory pattern should be prespecified as biological modifiers.

Sampling should cover physiologically distinct phases. A pragmatic design might include a standardized pre-anaesthetic baseline, an early post-anaesthesia or post-delivery measurement, a recording after initial mobilisation, and one or more measurements during the first postpartum days. The precise schedule should be driven by the hypothesized time course of autonomic and inflammatory change, not by convenience alone. Continuous wearable monitoring may complement standardized ECG epochs, but agreement between devices and susceptibility to motion artefact must be established before the signals are combined.

## From marker to perturbation: non-invasive autonomic modulation

Interventions can be used not only as treatments but also as mechanistic perturbations. Transcutaneous auricular vagus nerve stimulation (taVNS) stimulates auricular regions with vagal afferent innervation and may influence brainstem autonomic networks and inflammatory signalling. However, stimulation site, intensity, duty cycle, blinding, sham credibility and physiological target engagement vary across studies (Han et al., 2026). Demonstrating a change in HRV alone would not prove activation of the splenic anti-inflammatory circuit; concurrent inflammatory and clinical outcomes are required.

Slow-paced breathing alters respiratory sinus arrhythmia and may increase time-domain indices of vagally mediated HRV during the intervention. At resonance-frequency breathing of approximately 0.1 Hz, however, respiratory power shifts into the conventional LF band; LF and HF values obtained during paced breathing are therefore not directly comparable with spontaneous-breathing values, and HF power should not be interpreted in isolation (Laborde et al., 2022). This acute physiological effect must also be distinguished from sustained improvement in postoperative recovery. HRV biofeedback combines paced breathing with real-time feedback. In a randomized trial after caesarean delivery, HRV biofeedback increased SDNN and LF power and reduced stress and anxiety but did not significantly increase RMSSD or HF power in the overall sample (Chen et al., 2024). The findings support feasibility and selected psychological effects, not proof that cardiac parasympathetic modulation or inflammatory recovery was improved.

Future trials should therefore define whether the intervention is intended to modify cardiac autonomic regulation, inflammatory activity, symptoms or global recovery. A credible sham, blinded outcome assessment, standardized HRV acquisition and a prespecified mediation analysis would help distinguish target engagement from nonspecific effects of attention, breathing instruction or expectancy. In observational cohorts, temporally ordered mediation models could also test the hypothesized sequence of HRV-related autonomic change, inflammatory response and recovery, provided that temporal ordering and exposure-mediator and mediator-outcome confounding are prespecified; such analyses would not alone establish causality.

These non-pharmacological approaches are attractive in the postpartum setting because they avoid systemic drug exposure, but this advantage should not be confused with established effectiveness or universal acceptability. Ear discomfort, stimulation-related sensations, adherence, maternal workload and interference with infant care should be measured. Safety reporting should include bradycardia, dizziness, skin reactions and any treatment discontinuation, even when serious adverse effects are expected to be uncommon.

## Discussion

The proposed framework is physiologically coherent but clinically unproven. Its main strength is that it separates three related but non-equivalent constructs: cardiac autonomic modulation measured by HRV, central and peripheral autonomic signalling, and immune effector activity. Conflating these levels would turn a useful proxy into an unsupported mechanism.

Four uncertainties are central. First, the anatomy of the central-to-splenic relay remains contested and may differ across inflammatory states. Second, HRV is affected by respiration, heart rate and perioperative exposures, making standardization and sensitivity analyses essential. Third, the postpartum period contains biological modifiers that are not simply nuisance confounders, including lactation, uterine involution, sleep loss and pain. Fourth, current obstetric evidence is dominated by single preoperative measurements and hypotension prediction rather than longitudinal recovery phenotyping.

A rigorous validation study should combine short standardized ECG recordings with concurrent respiratory measurement, inflammatory biomarkers and clinically meaningful recovery outcomes at prespecified perioperative time points. Repeated-measures modelling should evaluate within-patient trajectories and account for baseline heart rate, mode and urgency of delivery, labour exposure, anaesthetic technique, blood loss, anaemia, medication and breastfeeding. Such a design could establish whether HRV adds incremental information beyond routine clinical variables and patient-reported outcomes.

The framework should be considered falsifiable. Failure of serial HRV to correlate with inflammation or recovery would argue against its inclusion in autonomic-immune recovery phenotyping, even if HRV remains useful for haemodynamic prediction. Conversely, a reproducible temporal association, especially if modified by a well-controlled autonomic intervention, would justify larger mechanistic trials.

Several analytical safeguards are important. Associations should be examined both before and after adjustment for heart rate and respiratory variables. Missingness caused by motion, ectopy or poor signal quality should be reported by time point and study group. Because HRV metrics are correlated, investigators should prespecify a primary autonomic measure and limit multiplicity. The clinical value of HRV should be judged by calibration, discrimination or incremental explanatory value, not only by statistically significant correlations.

This review is limited by its purposive rather than systematic literature approach and by the scarcity of studies that jointly measure autonomic, inflammatory and recovery outcomes after caesarean delivery. Much of the mechanistic evidence derives from endotoxaemia or animal models, while human perioperative studies are heterogeneous. The proposed framework should therefore be viewed as a structured research agenda rather than a summary of an established causal pathway.

## Conclusion

Vagal and related autonomic pathways may contribute to selected components of recovery after caesarean delivery, but the causal chain from cardiac HRV to splenic anti-inflammatory signalling has not been demonstrated. HRV is best positioned as one component of multimodal maternal recovery phenotyping. Its value will depend on standardized longitudinal measurement, direct comparison with inflammatory biomarkers and integration with patient-reported and clinical outcomes. taVNS, slow-paced breathing and HRV biofeedback provide plausible experimental probes, but efficacy and mechanism should be evaluated separately.
